# 

**Published:** 1908-04

**Authors:** 


### Journal-Record of Medicine

Successor to Atlanta Medical and Surgical Journal, Established 1855, and Southern Medical Record, Established 1870.

**OWNED BY THE ATLANTA MEDICAL JOURNAL CO.**

**Published Monthly.**

*Official Organ Fulton County Medical Society, State Examining Board, Presbyterian Hospital, Etc.*

EDGAR G. BALLENGER, M. D., Editor.
BERNARD WOLFF, M. D.. Supervising Editor.
A. W. STIRLING, M. D., C. M., D. P. H., JNO. S. HURT, M. D., Associate Editors.
GEORGE BROWN, M. D., Business Manager.

COLABORATORS

DR. W. F. WESTMORLAND, General Surgery.
F. W. McRAE, M. D., Abdominal Surgery.
H. F. HARRIS, M. D.. Pathology and Bacteriology.
E. B. BLOCK, M. D., Diseases of the Nervous System.
MICHAEL HOKE, M. D., Orthopedic Surgery.
CYRUS W. STRICKLER, M. D., Legal Medicine and Medical Legislation.
E. C. DAVIS, A. B., M. D., Obstetrics.
E. G. JONES, A. B., M. D., Gynecology.
R. T. DORSEY, Jr., B. S., M. D., Medicine.
J. N. LeCONTE, M. D.. Diseases of the Stomach and Intestines.
L. B. CLARKE. M. D., Pediatrics.
EDGAR PAULIN, M. D., Opsonic Medicine.
R. R. DALY, M. D., Medical Society.
A. W. STIRLING, M. D., Etc., Diseases of the Eye, Ear, Nose and Throat.
BERNARD WOLFF, M. D., Diseases of the Skin.
E. G. BALLENGER, M. D., Diseases of the Genito Urinary Organs.

LIBRARY
SURGEON GENERAL'S OFFICE
SEP.-7-1908
202659.

VOL. X. APRIL, 1908. No. 1

### NOTICE TO SUBSCRIBERS.

The Post Office Department, at Washington, has recently issued a regulation to which we desire to call the especial attention of some of our subscribers. The new ruling requires that all subscriptions to periodicals be paid in advance; that renewals must be subject to the same conditions; that only four months grace will be allowed monthly periodicals, and that if subscriptions or renewals are not paid within this time the journals to such subscribers will be denied the transmission in the mails at second-class rates.

We are left no discretion, as in the past, in extending credit to responsible parties, no matter how long they have been subscribers. So our readers will confer a great favor upon us and obviate an embarrassing situation if they will be so considerate as to make a settlement of past accounts and renew their subscriptions. An earnest effort will be made on our part to publish a journal well worth the subscription price, and with the increased editorial staff, to which we referred in our last issue, we believe that in the future the JOURNAL-RECORD will present much more original and valuable contributions than it has been able to do in the past.

The object of this action of the Post Office Department is to lessen the annual deficit, which is believed to be largely due to irregular periodicals flooding the mails with second-class matter which is not sent to legitimate subscribers.

We trust subscribers to the JOURNAL-RECORD who are delinquent will assist us in complying with the above requirements.

